# Public attitudes to population data research in 2022.

**DOI:** 10.23889/ijpds.v7i3.2019

**Published:** 2022-08-25

**Authors:** Shayda Kashef, Mary Cowan, Elizabeth Waind

**Affiliations:** 1 ADR UK; 2 Office for Statistics Regulation; 3 DARE UK

## Objectives

Broad public acceptability of research using data and statistics entails demonstrating trustworthiness and understanding public needs to maximise public benefit. Insight into public attitudes is crucial for informing how to operate in a trustworthy way and within the public interest.

## Approach

In 2022, two UK-wide public dialogues were undertaken to explore attitudes regarding the use of data and statistics in research. The first, explored views towards the creation of a more joined-up, efficient and trustworthy national data research infrastructure. The second aimed at understanding public perceptions of ‘public good’ of data and statistics. Both took a deliberative approach and involved diverse participation from members of the UK public. Both also sought the expertise of stakeholders within the data and statistics communities to inform the process.

## Results

The first dialogue found that the public want:

more proactive transparency around data research processes, with greater efforts made to raise awareness about it and the security processes in place;

meaningful and inclusive public involvement and engagement;

a more standardised, centralised and unified approach to data research across the UK; and

for sensitive data to be made safely and securely available to researchers for projects in the public benefit, regardless of whether those researchers sit within academia, government or the private sector.

The second dialogue launched in March; results will be publicly available by the middle of summer. The findings of this dialogue are intended to influence policy and process relating to the use of data and statistics.

## Conclusion

The first dialogue emphasises that the public are supportive of data research. And whilst reassured by security processes, they also do not want them to unduly hinder public benefit. Attendees at the IPDLN Conference will be some of the first to hear about the findings from the second dialogue.

